# Nature of the Virus Associated with Endemic Balkan Nephropathy

**DOI:** 10.3201/eid0808.020042

**Published:** 2002-08

**Authors:** Cristina Riquelme, David Escors, Javier Ortego, Carlos M. Sanchez, Branislava Uzelac-Keserovic, Konstantin Apostolov, Luis Enjuanes

**Affiliations:** * Campus Universidad Autónoma, Cantoblanco, Madrid, Spain; † Institute of Virology “Torlak,” Belgrade, Yugoslavia; ‡ Royal Postgraduate Medical School, London, United Kingdom

**Keywords:** Coronavirus, Balkan nephropathy

Endemic Balkan nephropathy (EBN), a disease restricted to three Balkan countries (Bulgaria, Rumania, and Yugoslavia), is characterized by a progressive shrinking of the kidneys and, in some cases, tumors in the proximal regions of the urinary tract *1*,*2*. A coronavirus was reported to be involved in the etiology of the disease, mostly on the basis of the isolation of a virus in cultures of kidney cells from a patient with EBN *1*,*3*. In addition, EBN-associated virus is reported to share serologic homology with human coronaviruses OC43 and 229E, as well as the porcine transmissible gastroenteritis coronavirus (formal name: *Transmissible gastroenteritis virus* [TGEV]), a virus that our laboratory has been studying for 16 years *4*,*5*. The objective of this commentary is to clarify whether the EBN-associated virus is in fact related to members of the *Coronaviridae* family *6*.

## Characterization of the Virus in EBN Primary Kidney Cell Cultures

The EBN-associated virus was isolated from primary kidney cells cultures, grown from fresh renal biopsy specimens of clinically confirmed cases of EBN *3*. The virus grown in the primary kidney cultures was used to infect Vero cells (ATCC CRL 1586) and sent to our laboratory for further identification.

A titration method was set up for the EBN-associated virus in Vero cells, as described for coronaviruses *7*. The virus had a small plaque phenotype and titers of 10^6^ to 10^7^ PFU/mL. No specific neutralization was observed when polyvalent or monoclonal antibodies that neutralized TGEV or the human coronaviruses OC43 or 229E were used in a standard neutralization assay. Furthermore, we observed no reactivity by immunofluorescence microscopy with the same antisera and specific monoclonal antibodies *7* on cells infected with the EBN-associated virus. In contrast, cell cultures infected with human coronaviruses or TGEV were positive with the corresponding antibodies.

Since coronavirus morphology is easily recognized by electron microscopy, Vero cells infected with the EBN-associated virus were embedded in resin for electron microscopy, and ultrathin sections were examined. Coronaviruses interacting with the cell membrane or inside the cell cytoplasm were easily seen when grown in swine testicle cells (Figure, A and C). Coronaviruses bud at the intermediate compartment and Golgi membranes (Figure, C) and the cytopathic effect on infected cells differed from that observed after infection by the EBN-associated virus. The most characteristic features of EBN-associated virus infection were the accumulation of stacked intracellular membranes and a general disorganization of the cytoplasmic membranous system (Figure, D). No apparent effect on the nucleus structure was observed. Electron-dense spherical virus particles approximately 30 nm in diameter were observed in the cytoplasm of infected cells but not in the nucleus. The size of these particles corresponds to that of the virions partially purified from the same cells. The particles appear to have an internal dense nucleocapsid (Figure, C). No viral factories were identified in association with the membranous structures. No viruslike particles were observed in uninfected Vero cells that resembled the virions described in the infected ones.

**Figure:**
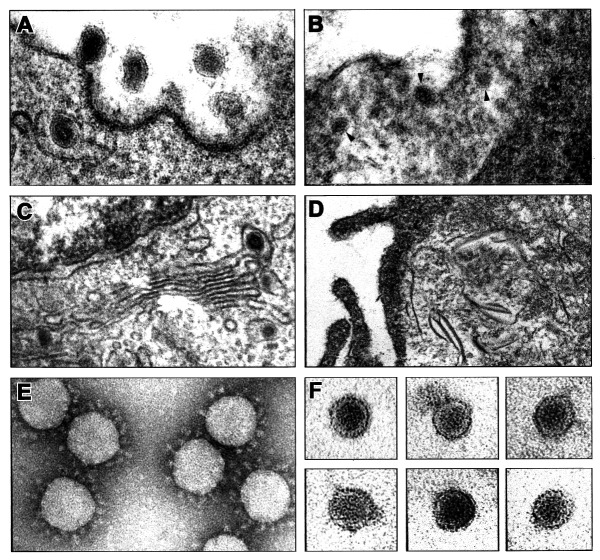
Electron microscopy images of thin sections and partially purified virions from cells infected with coronavirus or a virus tentatively associated with the endemic Balkan nephropathy (EBN). A and B. Electron microscopy images of thin sections of swine testicle cell infected with porcine transmissible gastroenteritis coronavirus (TGEV), showing virus binding to cell membrane at 8 h postinfection (A) or immature TGEV virions in the Golgi cisternae (C). B and D. Micrographs of thin sections of Vero cells infected with the virus tentatively associated with EBN at 12 h postinfection. B. The presence of EBN virions in the cytoplasm of the infected cells is indicated by arrows. D. Disorganization of the cytoplasmic membranous system in EBN-infected Vero cells. Electron microscopy images of concentrated TGEV (E) or EBN (F) virions negatively stained with 2% uranyl acetate. Bars in panels A–F represent 50 nm.

Supernatants of Vero cells infected with the EBN-associated virus were concentrated 100-fold by ultracentrifugation and visualized by negative staining with 2% uranyl acetate. Using purified TGEV as a standard, we observed only one type of spherical virion with a homogeneous mean virion size 28.4 nm ± 2 nm in diameter (coefficient of variation 7.1%; n=30) (Figure, F). In contrast, electron microscopy preparations of TGEV observed in parallel showed virions approximately 120 nm in diameter, with a corona of typical projecting peplomers *8*,*9* (Figure, C). The morphology of TGEV clearly differed from that of the EBN-associated virus that had no peplomers. The EBN-associated virion morphology and size were similar to that of small nonenveloped viruses such as picornavirus and parvovirus.

## Conclusion

The virus source used in these experiments is the same as that previously analyzed *3*, leading us to conclude that a coronavirus was present in the primary cell cultures from patients with the endemic nephropathy. The dominant and only virus detected in the cell cultures infected with the EBN-associated virus was unrelated to coronaviruses. Accordingly, we think that the involvement of a coronavirus should no longer be considered in EBN induction. Further studies are needed to clarify the nature of the 28.4-nm, non-enveloped virus particles found in the kidney cells of patients with EBN and to determine whether this virus is the causal agent of the disease.

## References

[1] Apostolov K, Spaic P. Evidence of a viral aetiology in endemic (Balkan) nephropathy. Lancet. 1975;2:1271–3. 10.1016/S0140-6736(75)90609-1 54796 PMC7135235

[2] Castegnaro M, Bartsch H, Chernozemsky I. Endemic nephropathy and urinary tract tumors in the Balkans. Cancer Res. 1987;47:3608–9.

[3] Uzelac-Keserovic B, Spasic P, Bojanic N, Dimitrijevic J, Lako B, Lepsanovic Z, Isolation of a coronavirus from kidney biopsies of endemic Balkan nephropathy patients. Nephron. 1999;81:141–5. 10.1159/000045269 9933748 PMC7179538

[4] Almazán F, González JM, Pénzes Z, Izeta A, Calvo E, Plana-Durán J, Engineering the largest RNA virus genome as an infectious bacterial artificial chromosome. Proc Natl Acad Sci U S A. 2000;97:5516–21. 10.1073/pnas.97.10.5516 10805807 PMC25860

[5] Enjuanes L, Siddell SG, Spaan WJ. Coronaviruses and arteriviruses. New York: Plenum Press; 1998.

[6] Enjuanes L, Brian D, Cavanagh D, Holmes K, Lai MMC, Laude H, Coronaviridae. In: van Regenmortel MHV, Fauquet CM, Bishop DHL, Carsten EB, Estes MK, Lemon SM, et al., editors. Virus taxonomy: classification and nomenclature of viruses. New York: Academic Press; 2000. p. 835–49.

[7] Sánchez CM, Jiménez G, Laviada MD, Correa I, Suñé C, Bullido MJ, Antigenic homology among coronaviruses related to transmissible gastroenteritis virus. Virology. 1990;174:410–7. 10.1016/0042-6822(90)90094-8 1689525 PMC7130632

[8] Enjuanes L, Spaan W, Snijder E, Cavanagh D. Nidovirales. In: van Regenmortel MHV, Fauquet CM, Bishop DHL, Carsten EB, Estes MK, Lemon SM, et al., editors. Virus taxonomy: classification and nomenclature of viruses. New York: Academic Press; 2000. p. 827–34.

[9] Escors D, Ortego J, Laude H, Enjuanes L. The membrane M protein carboxy terminus binds to transmissible gastroenteritis coronavirus core and contributes to core stability. J Virol. 2001;75:1312–24. 10.1128/JVI.75.3.1312-1324.2001 11152504 PMC114037

